# 894 Three Year Acinetobacter Spp. Infection Patterns in the Burn Intensive Care Unit

**DOI:** 10.1093/jbcr/iraf019.425

**Published:** 2025-04-01

**Authors:** Shannon Kuhrau

**Affiliations:** Loyola Burn Center

## Abstract

**Introduction:**

Patients with burns are at high risk of developing multi-drug resistant organisms which can contribute to poor wound healing, longer hospital lengths of stay, and mortality. Acinetobacter spp. infections can develop in this patient population and are often associated with some type of resistance leading to difficulties in treatment. The purpose of this study is to describe the characteristics, resistance patterns and eventual antibiotic selection in this difficult-to-treat infection in the burn population.

**Methods:**

Retrospective chart review of patients with Acinetobacter infections who were admitted to the burn intensive care unit at Loyola University Medical Center from September 1, 2021 through September 1, 2024. Patients were included if they ever grew Acinetobacter spp. in a tissue culture, blood culture, or bronchoalveolar lavage (BAL) culture. Baseline characteristics were collected and included total body surface area (TBSA) burned, mechanism of burn, presence of inhalation injury, type of grafting, and number of surgical operations. Infection data was collected including time to infection, type of sample, initial/final susceptibilities, and bacterial/fungal co-infections.

**Results:**

A total of 12 patients developed Acinetobacter spp. infections in the Burn ICU over a three-year time span. The median TBSA for patients was 32.5[IQR: 14-43.75]% with the most common mechanism of flame burn (58.3%). The most common location of infection was BAL (66.7%) closely followed by bloodstream infections (58.3%) and tissue infections (33.3%). A total of 5 patients (41.4%) had multiple sources of Acinetobacter spp. infection with 4 patients (33.3%) with subsequent culture positivity. Time to initial positive Acinetobacter spp. culture was 9 [5.5-13.3] days. Susceptibility patterns varied between patients with a total of 7 patients (58.3%) demonstrating resistance to at least one agent and 4 patients (33.3%) grew a carbapenem-resistant species. Bacterial co-infections and fungal co-infections occurred in 91.7% and 50% of patients respectively. In-hospital mortality occurred in 7 patients (58.3%) and median hospital LOS was 55 [24.8-69.2] days.

**Conclusions:**

Acinetobacter spp. infections presented most commonly with higher TBSA burns and was frequently found to be resistant to at least one agent. Clinical outcomes in this patient population remained poor which may have been influenced by the resistant infection.

**Applicability of Research to Practice:**

Resistance to Acinetobacter spp. infections is common. Optimal empiric antibiotic selection pending definitive susceptibility data has yet to be determined and this study can be utilized to help guide initial antibiotic management.

**Funding for the Study:**

N/A

